# Case Report: A Challenging Clinical Problem of Calcitonin-Negative Medullary Thyroid Cancer Diagnosis and Surveillance

**DOI:** 10.7759/cureus.32088

**Published:** 2022-11-30

**Authors:** Wania Rafaey, Asim Munir Alvi, Ahmed Imran Siddiqi, Waqas Shafiq, Hira Irfan

**Affiliations:** 1 Endocrinology, Diabetes and Metabolism, Shaukat Khanum Memorial Cancer Hospital and Research Centre, Lahore, PAK

**Keywords:** carcinoembryoinic antigen, thyroid nodule, calcitonin negative medullary thyroid carcinoma, medullary thyroid carcinoma, calcitonin

## Abstract

Medullary carcinoma of the thyroid is a rare neuroendocrine carcinoma that originates from the malignant proliferation of parafollicular C cells. In almost 100% of the cases, medullary carcinoma of the thyroid is associated with high levels of calcitonin and carcinoembryonic acid (CEA). Both carcinoembryonic antigen and calcitonin are used for the diagnosis and surveillance of medullary carcinoma of the thyroid. Calcitonin-negative medullary carcinoma of the thyroid is an extremely rare entity that is characterized by classic medullary carcinoma of the thyroid morphology without raised serum calcitonin levels. We describe the case of a middle-aged lady who presented with a few-month history of neck swelling associated with compressive symptoms. CT of the neck showed a large right thyroid nodule with central necrosis and retrosternal extension to the superior mediastinum. There was also a 360-degree encasement of the right common carotid artery. She underwent fine needle aspiration (FNAC) of the right thyroid nodule, and histopathology showed typical features of medullary carcinoma of the thyroid. Immunohistochemical staining for calcitonin and carcinoembryonic antigen was negative but positive for other neuroendocrine markers, i.e., synaptophysin and chromogranin A. Serum calcitonin and carcinoembryonic antigen levels were also in the normal range. So, a rare diagnosis of calcitonin-negative medullary carcinoma of the thyroid was made. As the disease was inoperable because of vascular encasement, a plan for external beam radiation therapy (ERBT) to the neck was made. Medullary carcinoma of the thyroid with normal serum levels of calcitonin is a very rare entity, with only a few cases reported in the literature.

In this case report, we have presented a rare case of medullary thyroid carcinoma (MTC) with normal-range serum calcitonin levels, how it was diagnosed, and how to follow up postoperatively.

## Introduction

Medullary thyroid carcinoma (MTC) is a neuroendocrine tumor that originates from the malignant proliferation of thyroid parafollicular C-cells; it represents 1-10% of all thyroid cancers. Parafollicular C-cells are derived from the neural crest ectoderm and ultimo branchial body and account for 1% of all thyroid cells. Parafollicular cells synthesize and secrete calcitonin, which regulates calcium homeostasis. The mean survival rate of patients with medullary thyroid carcinoma is 8.6 years, with the 10-year survival rate ranging from 69% to 89% [1-3]. In 75% of the cases, medullary thyroid carcinoma occurs sporadically; however, in 25% of the cases, the occurrence is hereditary and associated with a germline mutation of the RET proto-oncogene on chromosome 10 [4]. The familial form is generally termed multiple endocrine neoplasias (MEN). MEN 2A includes medullary thyroid carcinoma, hyperparathyroidism, and pheochromocytoma, and MEN 2B includes pheochromocytoma, mucosal neuromas, and intestinal ganglioneuromas. The sporadic form of MTC grows slowly, is well differentiated, and is locally aggressive. However, the familial form can invade adjacent organs and metastasize to lymph nodes, and the central compartment (levels IV to VI) is frequently involved, followed by levels II to V. It can also disseminate hematogenous to the liver, lungs, and bones and has a worse prognosis. In almost 100% of the cases, MTC is associated with high levels of calcitonin and carcinoembryonic acid (CEA). Both carcinoembryonic antigen and calcitonin are used for the diagnosis and surveillance of MTC.

## Case presentation

**Figure 1:**
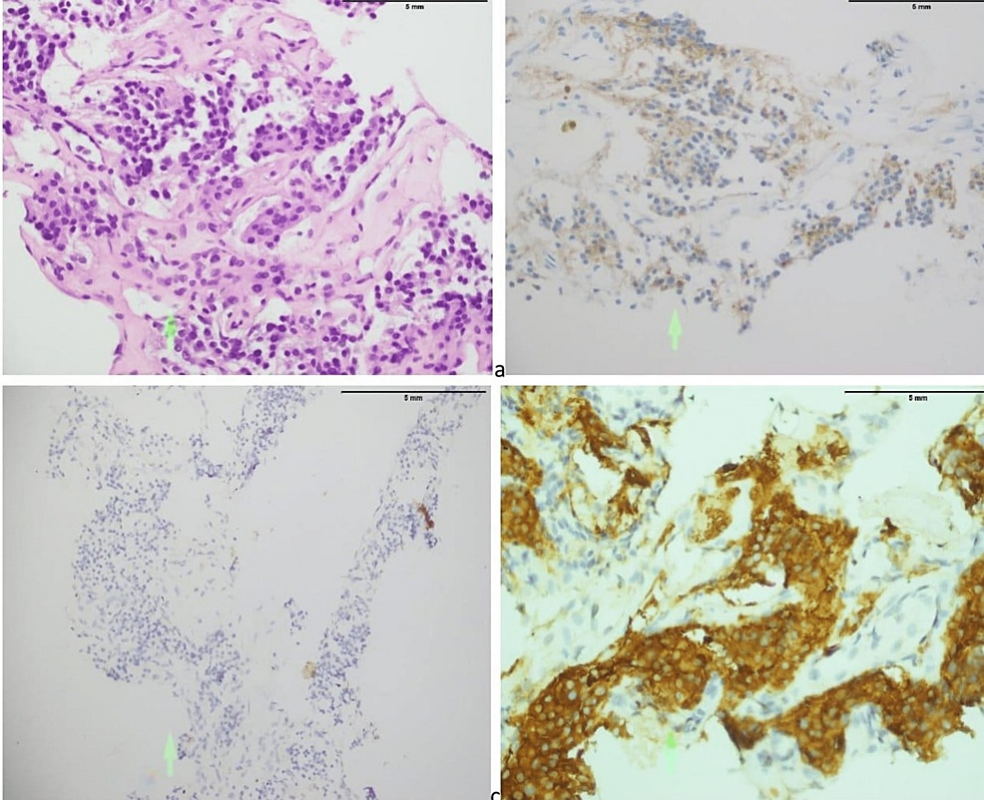
(a) Malignant neoplastic nests of atypical, plasmacytoid polygonal cells (green arrow); (b) tumor cells negative for calcitonin staining (green arrow); (c) tumor cells positively staining with chromogranin (green arrow); (d) tumor cells positively staining with synaptophysin (green arrow).

A 55-year-old Asian female with a known case of hypertension and no significant personal or family history of endocrine disorders presented with complaints of neck swelling and shortness of breath for the last few months. Shortness of breath used to aggravate on lying supine or in the left lateral position and used to get better on the right side. She had also been complaining of dysphagia, facial flushing, and increased perspiration, but no complaints of hoarseness of voice or diarrhea. On examination, there was a large swelling in the anterior part of the neck moving with deglutition. Ultrasound of the thyroid gland showed a hypoechoic nodule in the right lobe of the thyroid gland. A biopsy of the right thyroid lobe was performed, which revealed a single small piece of tissue infiltrated by the malignant neoplasm and showed nests of atypical round, plasmacytoid polygonal cells (Figure 1a), suggestive of medullary carcinoma of the thyroid. The immunohistochemical stain was calcitonin negative (Figure 1b), chromogranin positive (Figure 1c), and synaptophysin positive (Figure 1d).

**Figure 2:**
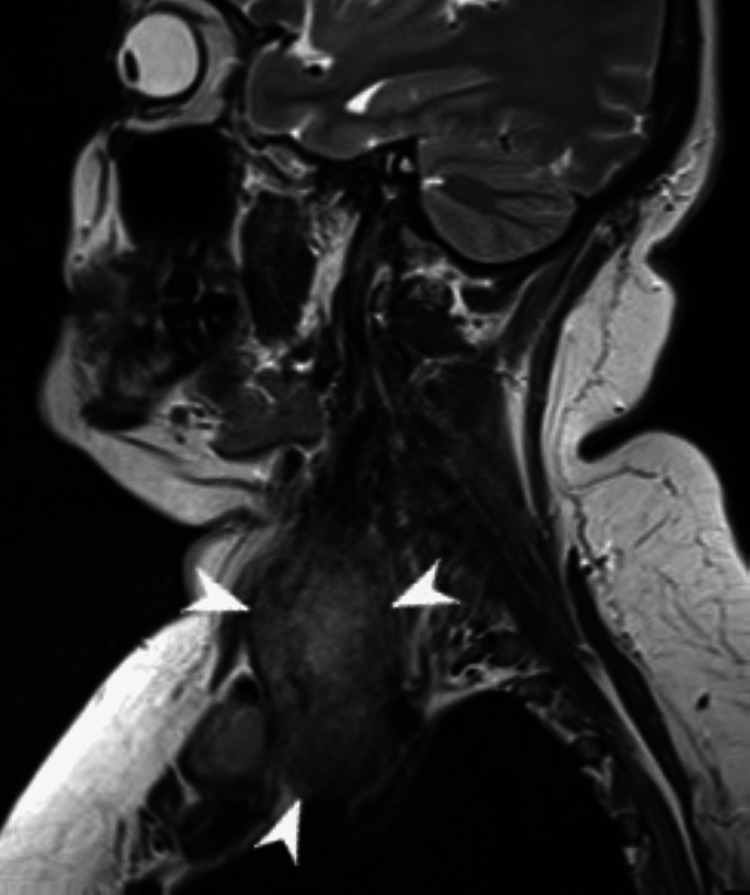
MRI neck without contrast images demonstrating a large heterogeneous mass arising from the right thyroid lobe.

MRI of the neck without contrast showed a large heterogeneous soft tissue mass arising from the right thyroid that measures up to 6.5 cm in transverse diameter and 8.5 cm craniocaudally (Figure 2). The mass was displacing the trachea to the contralateral left side. Though the anterior thyroid capsule was intact, there was extra-thyroid disease on the lateral side. There was also the involvement of the strap muscles of the neck over the right lateral aspect superiorly. It was abutting the vascular structures, compressing SVC, encasement of carotid and B/L enlarge cervical LN levels II and III. CT of the chest, abdomen, and pelvis with contrast was unremarkable for hepatic, pulmonary, or nodal metastasis. A blood workup showed a normal thyroid profile, plasma metanephrines, normetanephrine, calcium, vitamin D, and PTH intact. Calcitonin was <2 pg/ml (0-5), and CEA was also within normal limits.

**Figure 3:**
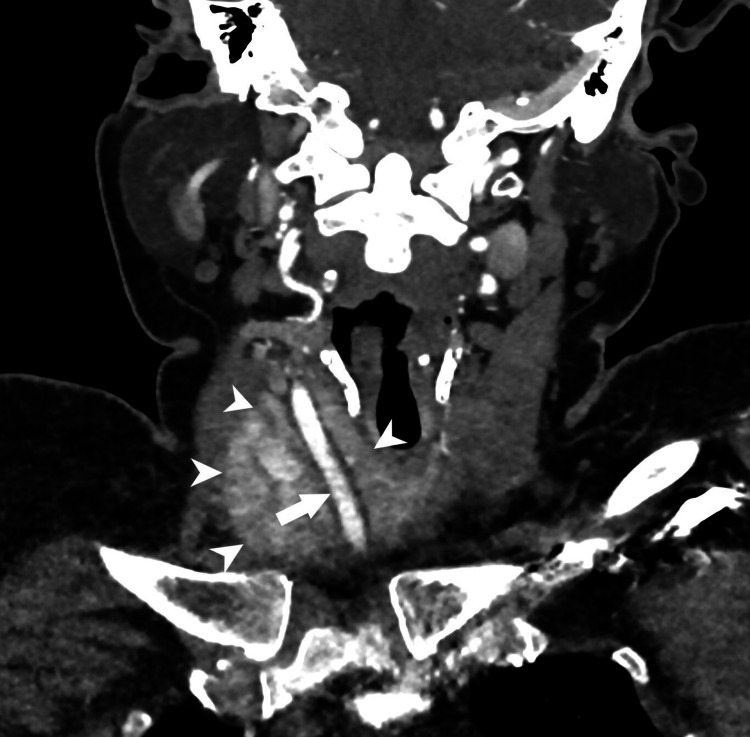
CT angiogram demonstrating large centrally necrotic nodal mass encasing right common carotid artery.

A CT angiogram showed a large, centrally necrotic nodal mass at station level VII, with extension up to the supraclavicular region and inferiorly reaching up to the superior mediastinum. There was a 360-degree encasement of the right common carotid artery, approximately 10 mm below the bifurcation (Figure 3). It showed irresectable disease. Palliative radiation therapy was given to gross disease mediastinum and neck for 20 Gy in 5 fractions of rapid arc. A follow-up ultrasound of the thyroid showed an interval decrease in the size of the right thyroid lobe.

## Discussion

Medullary thyroid carcinoma is a neuroendocrine tumor that originates from the malignant proliferation of thyroid parafollicular C-cells; it represents 1% to 10% of all thyroid cancers. Parafollicular C-cells are derived from the neural crest ectoderm and ultimo-branchial body and account for 1% of all thyroid cells. Parafollicular cells synthesize and secrete calcitonin, which regulates calcium homeostasis. The mean survival rate of patients with medullary thyroid carcinoma is 8.6 years, with the 10-year survival rate ranging from 69% to 89% [1-3]. In 75% of the cases, medullary thyroid carcinoma occurs sporadically; however, in 25% of the cases, the occurrence is hereditary and associated with a germline mutation of the RET proto-oncogene on chromosome 10 [4]. The familial form is generally termed MEN. MEN 2A includes medullary thyroid carcinoma, hyperparathyroidism, and pheochromocytoma, and MEN 2B includes pheochromocytoma, mucosal neuromas, and intestinal ganglioneuromas. The sporadic form of medullary thyroid carcinoma grows slowly, is well-differentiated, and is locally aggressive. However, the familial form can invade adjacent organs and metastasize to lymph nodes; the central compartment (levels IV to VI) is frequently involved followed by levels II to V. It can also disseminate hematogenous to the liver, lungs, and bones and has a worse prognosis.

Parafollicular cells of the thyroid synthesize and secrete calcitonin, which is a highly sensitive and specific biochemical marker for early diagnosis, postoperative monitoring, and disease recurrence in patients with medullary thyroid carcinoma [5]. Serum calcitonin levels usually correlate with tumor size, volume, and disease burden. Raised basal serum calcitonin levels (>100 pg/ml) and after pentagastrin stimulation testing (>1000 pg/ml) are highly suggestive of medullary thyroid carcinoma. Medullary thyroid carcinoma, like other neuroendocrine tumors, also produces pro-calcitonin, the precursor of calcitonin, chromogranin A (Cg A), and neuron-specific enolase (NSE). Falsely high or low levels of calcitonin can be seen in other diseases as well, including autoimmune thyroiditis, C-cell hyperplasia, end-stage renal disease (ESRD), lung and prostate cancer, and other neuroendocrine disorders.

The pathophysiology of calcitonin-negative medullary thyroid carcinoma (CNTMTC) is less clearly understood. Some studies suggest that calcitonin-negative medullary thyroid carcinoma is due to dedifferentiation of the tumor cells, the production of aberrant calcitonin precursors not recognized by the testing antibodies, or the hook effect. The hook or prozone effect is the falsely low observed value when there is a large amount of analyte in the sample [6]. In rare cases, mutations of calcitonin gene-related peptide (CGRP) have been described as a cause of medullary thyroid carcinoma with normal serum calcitonin levels.

There are only a few cases of medullary thyroid carcinoma in the literature that are associated with normal calcitonin and CEA. They are called non-secretory medullary thyroid carcinoma or CTNMTC. Our case also presented histopathological features of medullary thyroid carcinoma but with normal calcitonin and CEA levels. The most characteristic features of medullary thyroid carcinoma are hypocalcemia and diarrhea to a lesser extent. This case had normal calcium levels and no complaints of diarrhea. The diagnosis of medullary thyroid carcinoma cannot be excluded even if serum calcitonin levels are normal. Another biochemical marker in the evaluation of calcitonin-negative medullary thyroid carcinoma is a CEA, whose increased preoperative levels are associated with medullary thyroid carcinoma progression and prognosis. Procalcitonin in the serum and calcitonin in the washout fluids of fine needle aspiration (FNAC-CT) are other specific tests for medullary thyroid carcinoma. However, FNAC and histopathology are the gold standard approaches in the evaluation and management of all thyroid nodules. Although FNAC has a lower diagnostic value for medullary thyroid carcinoma than any other differentiated thyroid malignancy, the presence of micro-calcifications and amyloid substances separated by fibrous septa are histological characteristics of medullary thyroid carcinoma [3,7]. In the current case as well, histopathology was suggestive of medullary thyroid carcinoma and biochemical markers were negative.

The preferable treatment option for patients with calcitonin-negative medullary thyroid carcinoma is surgery if the disease is localized; therefore, early diagnosis of the disease is the most significant prognostic indicator of the disease. However, when the disease is not resectable, other treatment options include systemic therapy or radiation therapy. In our case, since the disease was irresectable, external radiation beam therapy (ERBT) was given to the patient.

The main challenge in the management of patients with calcitonin-negative medullary thyroid carcinoma is the surveillance of the disease since the tumor markers, including calcitonin and other markers like CEA and chromogranin A, are not elevated. In these cases, procalcitonin and calcitonin gene-related peptide (CRGP) concentrations can be measured postoperatively [8]. There are several imaging techniques for the localization of disease recurrence: ultrasound neck, CT scan, MRI, and fluorodeoxyglucose positron emission tomography (FDG-PET). A follow-up ultrasound of the thyroid had been done in our case, and there was an interval decrease in the size of the right thyroid lobe mass. Patients with poorly differentiated medullary thyroid carcinoma are at higher risk of recurrence and mortality; therefore, a more extensive postoperative surveillance plan is required in these cases.

In conclusion, medullary thyroid carcinoma is a rare thyroid malignancy, and calcitonin-negative medullary thyroid carcinoma is even rarer. Its etiology is still debatable. Histopathology and immunohistochemistry studies are very important tools, as tumor markers are indispensable to arriving at a diagnosis. Postoperative surveillance with imaging modalities can be done until assays of tumor markers, procalcitonin, and CRGP are easily available.

## Conclusions

Medullary thyroid carcinoma is a rare thyroid malignancy, and calcitonin-negative medullary thyroid carcinoma is even more rare. Its etiology is still debatable. The normal level of calcitonin and CEA does not rule out medullary carcinoma, and the possibility of calcitonin-negative medullary thyroid carcinoma should be considered in suitable patients. Histopathology and immunohistochemistry studies are very important tools, as tumor markers are indispensable to arriving at a diagnosis. Surveillance of calcitonin-negative medullary thyroid carcinoma should be done with imaging techniques for the localization of disease recurrences such as ultrasound neck, CT-scan, MRI, and FDG-PET till assays of tumor markers, procalcitonin and CRGP are easily available.
